# Community Advisors’ Effect on a Randomized Pragmatic Clinical Trial for Asthma Treatment: Retrospective Analysis

**DOI:** 10.2196/84679

**Published:** 2025-12-19

**Authors:** Wilson D Pace, Barbara Yawn, Nancy E Maher, Brianna Ericson, Elizabeth W Staton, Paulina Arias Hernandez, Bonnie Telon Sosa, Jean Kruse, Elliot Israel

**Affiliations:** 1 DARTNet Institute, 12635 East Montview Boulevard, Mail Stop 3, Aurora, CO, 80045, United States, 1 (800) 434-0278; 2 Department of Family and Community Health, University of Minnesota, Minneapolis, MN, United States; 3 Division of Pulmonary and Critical Care Medicine, Brigham and Women’s Hospital, Boston, MA, United States; 4 Department of Internal Medicine, Allergy/Immunology Section, University of Puerto Rico: Medical Sciences Campus, San Juan Puerto Rico, United States Minor Outlying Islands

**Keywords:** asthma, patient involvement, participatory research, rescue inhaled corticosteroids, protocol impact

## Abstract

**Background:**

Community advisors including patients, families, clinicians, and payers are important partners who can guide clinical research; yet, there is little evidence documenting the impact of community engagement on study changes and outcomes.

**Objective:**

This study aims to describe the effect of community advisor input on 2 concerns identified during the conduct of a clinical trial.

**Methods:**

Using data from the PREPARE (Person Empowered Asthma Relief) randomized clinical trial studying the use of inhaled corticosteroid (ICS) as part of rescue therapy for asthma, we examined the effect of protocol changes suggested by community advisors to address study implementation concerns.

**Results:**

Community advisors addressed 2 issues that threatened the success of the research: low response rates to monthly outcome surveys and low reported use of ICS with nebulizer rescue treatments. Initial low survey response rates were addressed by changing reminder frequency, shortening the survey, reducing the burden of logging in, and adding a raffle prize for timely responses. In the pilot phase of the study, the overall 3-month survey response rate was 67% (64 completed of 96 possible surveys). After protocol changes, the survey response rate over the first 3 months was 96.08% (3404 completed of 3543 possible surveys) and was 87.38% (1032 of 1181 participants) for each individual’s final 3 months; the overall response rate for the full study was 92.3%. For the full study, 72.1% (n=850) of 1181 participants completed all of their first three surveys compared with only 25% (n=8) of 32 pilot enrollees. Early low use of ICS with nebulizers was addressed by additional communication, reminder stickers, and designing a method to attach a provided ICS inhaler to the nebulizers. The percentage of people reporting use of 3 to 5 puffs of ICS with each nebulizer treatment rose from 42.1% (200/475) in the early full study to 75.4% (525/696) following the protocol changes.

**Conclusions:**

Multicomponent changes to the PREPARE protocol crafted by community advisors were associated with improved monthly survey rates and ICS adherence during nebulizer use.

## Introduction

Input of community advisors at all stages of clinical research has become a frequent expectation of many funders and research agencies over the past decade. Community advisor engagement, also known as stakeholder engagement or patient and public involvement, can consist of engaging a broad variety of individuals including patients, caregivers, practicing clinicians, patient or condition advocates, professional societies, policy experts, payers, and others. In the United States, this advance has been championed by the Patient-Centered Outcomes Research Institute (PCORI) [1]. The construct of “stakeholder” engagement has been adopted by US federal agencies; for instance, the Centers for Disease Control and Prevention recommends, “**Involve stakeholders in key activities** throughout the planning and implementation” of the activity [2]. A similar emphasis can be found in selected funding opportunity announcements from The Agency for Healthcare Research and Quality [3], the Food and Drug Administration [4], and the National Institutes of Health [5,6]. Internationally, the World Health Organization [7] and the National Institute for Health Research of the United Kingdom [8] have included the need for “stakeholder” input within funding announcements. Furthermore, patients and families have called for increased transparency, particularly in clinical trials registries, about whether the trials involved patients in the design process [9]. Community advisor engagement has long been an important component within various medical research constructs. Practice-based research networks (PBRNs) [10], particularly in primary care, consider clinician input and vetting of all projects as a critical step. Furthermore, a 2005 survey of primary care PBRNs found that many had also engaged community members to help set their research agenda, review research protocols, and help interpret and report the results of studies [10]. Community-based participatory research starts the research process with a community assessment and broad-based input from community groups [11,12].

Despite the increased focus on community advisor engagement and demand from several funders, the evidence base for community advisor impact is fairly limited. Research on the impact of community advisor engagement has primarily focused on recruitment and retention of research participants. A review of the literature in this area indicates a small positive impact on recruitment (odds ratio [OR] 1.16, 95% confidence interval and prediction interval 1.01‐1.34), with increased impact when studies include people with a lived experience when designing recruitment approaches (OR 3.14, 95% confidence interval and prediction interval 1.89‐5.22) [13]. The impact on retention was inconclusive. Concannon et al [14] call for evaluative research in community advisor engagement to include its impact on relevance, transparency, and adoption of research [14]. In her review of the area, Esmail et al [15] focused on the community advisors’ impact on transparency during recruitment, dissemination, and uptake of results. None of these reviews, nor an accompanying editorial from PCORI [16], found information on or assessed the impact of community advisor engagement in the ongoing operations of a research project.

The PREPARE (Person Empowered Asthma Relief) study assessed the effect of using inhaled corticosteroid (ICS) as part of asthma rescue therapy [17]. PREPARE engaged a wide variety of community advisors from the conceptualization of the project, throughout the execution of the study, and during the interpretation and reporting of the results. This paper reports on the impact of community advisor input on 2 specific issues. Both issues for this post hoc analysis were identified during the conduct of the pragmatic clinical trial: lower than expected survey response rates and lower than expected medication use.

## Methods

### The PREPARE Study

The PREPARE study was a patient-randomized, open-label, pragmatic clinical trial comparing the use of patient-activated, reliever-triggered inhaled corticosteroid therapy added to a person’s usual quick-acting or “rescue” asthma therapy while continuing all other usual asthma care. The study methods [18] and outcomes [17] have been described elsewhere. The PREPARE study was guided by extensive community advisor input, specifically patient advisors (1 English-speaking group and 1 Spanish-speaking group), a clinical site advisor group, a scientific advisory group, and a group of professional society or patient advocacy and policy representatives. An executive committee, which oversaw and confirmed all study decisions, included representatives of each community advisor group as well as central research team members.

The 1201 participants in the PREPARE study were self-reported African American or Black (n=603) or Hispanic or Latinx (n=598) adults with asthma who reported previously being prescribed daily ICS as part of their controller therapy, recruited from 19 study sites across the United States. All participants demonstrated that they had their controller ICS on hand at their enrollment visit, though they did not need to be regular users of their prescribed ICS. In addition, each participant either had an Asthma Control Test score of <20 or in the past 12 months had an asthma exacerbation treated with systemic corticosteroids or was hospitalized. Intervention participants were asked to use 1 puff of beclomethasone (80 mcg, donated by Teva Pharmaceuticals) delivered via a metered dose inhaler whenever they used a puff of their rescue inhaler (typically a short-acting beta-agonist) or 5 puffs of the ICS inhaler whenever they used a nebulizer for rescue therapy. Outcomes were assessed through monthly online or telephone patient surveys collected over the 15-month-per-participant enrollment period. Surveys queried about exacerbation-like events, which were blindly adjudicated using medical record data or via direct patient interviews. The primary outcome for PREPARE was the difference in the annualized exacerbation rates between the intervention and control arms, highlighting the importance of high survey response rates. The full study was preceded by a Vanguard or pilot phase involving 4 clinical sites and 33 participants who were asked to follow all study activities for 3 months. The Vanguard outcomes have been previously described [19].

At the end of the Vanguard phase, as well as intermittently through the conduct of the full study, the research team presented concerns or issues to the relevant community advisor groups (often all of the groups). The community advisor groups held iterative discussions and asynchronous interactions to recommend solutions for identified concerns. Herein, we present 2 specific concerns as evidence of the impact of community advisor involvement on the research protocol.

During the Vanguard phase, the monthly survey response rate was lower than expected (64 completed of 96 possible surveys, 67%) and below the response rate on which the main study was powered (75%). During the main study, a second concern was the high self-reported rates of never using ICS concomitant with rescue nebulizer inhaler use (9.9%) and the low average number of self-reported puffs of ICS accompanying each nebulizer “rescue” treatment (42.1% ≥3 puffs) compared to the recommended 5 puffs of ICS with each nebulized rescue therapy use. The community advisors suggested adjustments to the study procedures to address these concerns, and the final protocol changes and their impacts are presented in the “Results” section to illustrate the importance of community advisor involvement. The community advisor-suggested changes were implemented in 2 steps between October 2018 and January 2019. Patients enrolled prior to December 2018 were considered not to have received the advisor-recommended interventions for analysis purposes. Patients enrolled between December 1, 2018, and January 31, 2019, were excluded from analysis during the implementation of the full set of advisor-recommended interventions. Patients enrolled after January 31, 2019, were considered to have received the revised approach for promoting ICS use with a nebulizer at enrollment.

The Fisher exact tests for small cell sizes were used to compare the monthly survey response rates between the Vanguard and full study time periods as well as for comparing the rates of nonuse of ICS with a nebulizer treatment. Chi-square tests were used to compare the level of ICS use before and after the intervention. A Bonferroni adjustment was used to determine the level of significance for multiple comparisons with 5 comparisons for the survey analysis (*P*<.01) and 4 comparisons for the ICS use analysis (*P*<.012). All analyses were conducted with SAS software (version 9.4; SAS Institute).

### Ethical Considerations

The PREPARE study was approved by the institutional review board of Partners HealthCare (2016P001839) and local institutional review boards. All enrolled participants provided written informed consent prior to any data collection. Any data collected with identifiers were obtained and stored using standard data protection methods approved by our institutional review boards and privacy officers. Advisors were research team members and participated voluntarily with reimbursement for their travel expenses. Advisors were paid US $75 per hour for committee work as well as for their meetings (including when they attended in-person meetings, usually 6 h in length). All advisors were paid the same rate, whether they were professional advisors or patient or caregiver advisors.

## Results

The Vanguard and full study population characteristics are shown in Table 1. ICS use with nebulizers was only possible in the intervention arm of the study; thus, this cohort is listed as an additional column in Table 1 related to the concern of low ICS use with rescue nebulizer therapy. The Vanguard patients were similar in age to the full study cohort, had a similar pattern of cigarette use, a similar history of exacerbations in the previous year, and similar health literacy risk based on baseline assessments. The intervention arm of the study closely matched the control arm, as demonstrated in the main effects paper [17] (data not presented here), as expected due to within-site, stratified randomization.

**Table 1.** Characteristics of the Vanguard and full study populations.

Characteristic	Vanguard (n=33)	Black (n=18)	Latinx (n=15)	Full study (n=1201)	Black (n=603)	Latinx (n=598)	Intervention arm (n=600)
Age (y), mean (SD)	47.2 (12.5)	43.8 (13.3)	51.3 (10.5)	47.7 (13.7)	48.1 (13.4)	47.2 (14.0)	48.3 (13.5)
Sex, female, n (%)	25 (76)	13 (72)	12 (80)	1005 (83.68)	508 (84.2)	497 (83.1)	508 (84.7)
Smoking status, n (%)							
Nonsmoker	25 (76)	16 (89)	9 (60)	958 (79.77)	469 (77.8)	489 (81.8)	477 (79.5)
Former	4 (12)	1 (6)	3 (20)	100 (8.33)	48 (8.0)	52 (8.7)	54 (9.0)
Current	4 (12)	1 (6)	3 (20)	143 (11.90)	57 (9.5)	57 (9.5)	69 (11.5)

The PREPARE study engaged 16 patient advisors in 2 languages, 42 clinical site advisors (site principal investigators and study coordinators), and another 22 individuals as scientific, advocacy, professional society, and policy community advisors. The patient partner advisors were all self-identified as African American or Black or Hispanic or Latinx or both and either had asthma or were caregivers of individuals with asthma or both. Patient and caregiver advisors were recruited from individuals receiving care at or otherwise affiliated with the primary research site (3 people), one of the clinical research sites (10 people), or from previous involvement with the research team (1 person). Other community advisors were selected to provide input from advocacy groups (eg, American Lung Association), professional societies (eg, American Academy of Asthma, Allergy and Immunology), health care systems, health care payers, both from the membership perspective and the pharmacy perspective (eg, a former state Medicaid director, senior staff within private insurance companies and Centers for Medicare and Medicaid Services, as well as individuals involved in health policy issues, and scientific experts). These individuals were appointed by the advocacy groups and professional societies. The payer and policy individuals were recruited through previous work with a study team member or from secondary recommendations from other advisors. The payer or policy advisors worked or had previously worked in their relevant roles at the state, federal, regional, or national levels. There was very little turnover across the advisory groups during the project; several patient or caregiver members dropped out, and two of the policy or payer advisors had to be replaced. These groups met independently, in ad hoc combinations, and all together at various times throughout the project.

Over the course of the project (primarily in the first year), community advisors were provided educational meetings, conversations, and slide decks concerning various aspects of the project. These activities included several sessions on the basics of our current understanding of asthma pathophysiology and treatment, several sessions on research methods, and sessions to promote group cohesion. All the sessions were voluntary and recorded for future review by interested advisors. While these presentations were well attended and sometimes offered more than once, they were by no means universally attended by all advisors. Overall, the sessions were more focused on supporting the patient and caregiver advisors than the advocacy, professional society, and payer or policy advisors.

**Figure 1.**
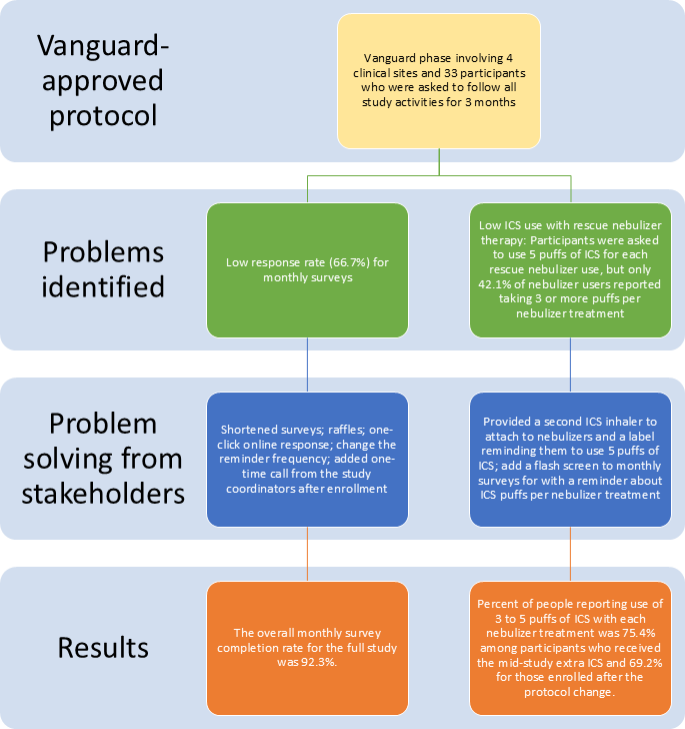
PREPARE (Person Empowered Asthma Relief) trial protocol information flow: problem identification, problem solving, and results. ICS: inhaled corticosteroid.

All community advisor groups considered the response rate and ICS use concerns and both independently and jointly developed adjustments to the study protocol to attempt to address the concerns. Details for the development of each protocol change are presented separately and summarized in Figure 1. Final study protocol adjustments were presented to the scientific committee and the executive committee for final approval and approved by the institutional review board of Partners HealthCare and local institutional review boards.

The first of the two issues presented is the initial low monthly survey response rates during the Vanguard phase. The results were a major concern as they were below the rates on which the study sample size calculations were powered. The 67% response rate (64 of 96 possible surveys) and the original methods used to support responses were shared with all community advisors at a face-to-face study meeting in June 2018, during which small groups, each of which included central research staff, site research staff, patients, and other community advisors, met and considered approaches to improving response rates. The patient advisors were instrumental in developing the initial recommendations, numbered 1 to 6 below. The approaches were presented to the full group and refined in an open discussion. After the face-to-face meeting, the proposed changes to the data collection protocol were circulated back to the patient advisor groups (1 English-speaking and 1 Spanish-speaking), then the clinical sites, and the professional society or patient advocacy and policy representatives. Responses and further refinements were collated and sent back to each group for their final approval. Six changes to the protocol were agreed upon by all groups and accepted by the scientific and executive committees. The six changes were: (1) shorten the monthly survey; (2) allow people to respond online without having to log in to the survey system; (3) change the reminder frequency from 28, 35, and 42 days to 28, 30, and 32 days around the 30-day due date; (4) remind people of each survey using three modalities (text, email, and voice) instead of just the individuals’ stated preferred method; (5) add a one-time call from the study coordinators after enrollment, at day 26 to 28, to remind participants of the monthly surveys and the importance of completing them; and (6) add a monthly raffle for people who responded within ±6 days of the 30-day due date (three people received an extra $100 each mo)—Florida residents were excluded by state law from the raffle.

The comparison between the full study and Vanguard monthly survey response rates is shown in Table 2. For the full study, the response rate over the first 3 months was 96.08% (3404 surveys completed of 3543 possible) and was 87.38% (1032 participants of 1181 participants) for each individual’s final 3 months; the overall response rate for the full study was 92.3% [17]. Both are significantly above the overall 3-month Vanguard response rate of 67%. For the full study, 72.1% (850) of 1181 participants completed all of their first three surveys compared with only 25% (8) of 32 of Vanguard enrollees.

**Table 2.** Comparison of monthly survey response rates between the full study and Vanguard.

Group	Vanguard (n=32), n (%)	Full study (n=1181), n (%)	*P* value (Fisher exact test for small cell counts)
Total possible surveys in first 3 months	96	3543	N/A^b^
Total surveys completed in first 3 months within 15 days prior to or 15 days after each month’s due date	64 (67)	3404 (96.08)	<.001
Total surveys completed in first 3 months within 6 days prior to or 6 days after each month’s due date	39 (41)	3176 (89.64)	<.001
Participants who completed **all surveys** first 3 months (month 1+month 2+month 3) within 15 days prior to or 15 days after each month’s due date	8 (25)	1072 (90.77)	<.001
Participants who completed **all surveys** first 3 months (month 1+month 2+month 3) within 6 days prior to or 6 days after each month’s due date	5 (16)	913 (77.31)	<.001
Participants who completed **last 3 expected^a^** surveys within 15 days prior to or 15 days after each month’s due date	8 (25)	1032 (87.38)	<.001

a For the full study, the last 3 expected surveys are usually month 13, month 14, and month 15 but due to recruitment timing, some participants did not complete all 15 surveys. Their “final expected” surveys are the last 3 expected prior to study end on April 30, 2021. For the Vanguard pilot study, the first 3-month and last 3-month surveys are the same surveys since the pilot only had 3 total surveys.

b N/A: not applicable.

The second issue identified related to the use of ICS with nebulizer treatments. After enrollment of the first 259 intervention participants (December 2018) in the full study, it was noted that over 60% of intervention participants used a nebulizer for rescue therapy some of the time, and over 40% used a rescue nebulizer on a weekly basis. Per the study protocol, intervention participants were asked to use 5 puffs of ICS for each rescue nebulizer use. Self-reported levels of any use of the ICS with a nebulizer were 90.5%, while self-reported use of 3 or more puffs was 42.1% (200/475) using the original study protocol.

Patient, clinical site, and professional society advisors were asked to consider options to improve these rates. The groups met individually by telephone or video meetings. All groups provided suggestions. These suggestions were collated and sent back to each group for further refinement. The final set of recommendations was approved by each of the advisory groups as well as the scientific and executive committees. The following adjustments were proposed:

1. Provide nebulizer users with a second ICS metered dose inhaler with a Velcro band to attach to their nebulizer.
2. Add a label to their nebulizer reminding them to use 5 puffs of ICS with each nebulizer treatment.
3. Send all nebulizer users already enrolled an extra ICS with the label and Velcro band.
4. Add a splash screen at the end of the monthly surveys for each group with a reminder. Usual care participants were reminded to take their controller medications regularly. Intervention participants were reminded to use ICS as part of rescue therapy and specifically to use 5 puffs when using a nebulizer.

The fourth recommendation was discussed with the funder and researchers involved with developing the Pragmatic Explanatory Continuum Indicator Summary 2 [20] coalition. Both indicated that the extra messaging was not a major concern within the framework of a pragmatic trial since such instructions would be given during usual clinical care.

Scientific committee advisors reviewed the changes and helped craft 3 differing letters to be sent to the control patients, the intervention participants who did not use rescue nebulizers, and the intervention participants who reported rescue nebulizer use, so that all active participants were contacted at the same time that the current intervention group nebulizer users were sent a second ICS inhaler. Individuals enrolled after this adjustment to the protocol (February 2019) who indicated that they used a nebulizer at intake were provided 2 ICS inhalers at enrollment, the Velcro band, and the message for their nebulizer if they were randomized to the intervention arm.

Table 3 shows the use of at least 3 to 5 puffs of ICS with nebulizer use before and after the community advisor-recommended changes. Following the intervention, the percentage of people reporting use of 3 to 5 puffs of ICS with each nebulizer treatment rose from 42.1% (200/475) to 75.4% (525/696) in the participants who received the mid-study extra ICS. This higher rate persisted with patients who were enrolled after the protocol change and received a second ICS inhaler, extra band, and reminders at the time of enrollment (458/662, 69.2%, for this group specifically). The percentage of individuals indicating no use of ICS with a nebulizer treatment at the monthly survey level decreased from 9.5% (45/475) to 4.6% (32/696*; P*=.006) for individuals enrolled prior to December 2018. The percentage of no ICS use in individuals enrolled after the new approach was instituted was 7.7% (51/662; not significant).

**Table 3.** Changes in reported inhaled corticosteroid (ICS) adherence and nonuse by study enrollment date.

Group	A: recruited prior to December 2018, surveys completed prior to December 2018 (n=475), n (%)^a^	B: recruited prior to December 2018, surveys completed after February 2019 (n=696), n (%)	C: recruited after February 2019, provided 2 ICS at intake (n=662), n (%)^a^	*P* value (A vs B)	*P* value (A vs C)
Surveys indicating QVAR RediHaler adherence (3+ puffs)	200 (42.1)	525 (75.4)	458 (69.2)	<.001	<.001
Surveys indicating nonuse of ICS (none of the time)	45 (9.5)	32 (4.6)	51 (7.7)	.006	.230

a Only months in which a participant indicated they used a nebulizer one or more times included the questions of interest for this analysis.

## Discussion

### Principal Findings

While the engagement of multiple community advisors in planning, conducting, and interpreting research is now frequently promoted and has strong face validity, there has been little evidence that community advisor involvement changes or affects research activities outside of recruitment [13-15]. This paper highlights the major positive changes observed after recommendations from various community advisors were implemented in 2 areas of concern for a large pragmatic clinical trial.

The PREPARE study engaged 80 or more people as community advisors, in 2 languages, with multiple perspectives on health care both received and delivered. This large number of people often engaged in synergistic discussions during meetings, with comments building upon others’ statements. We believe community advisors are more effective with a critical mass of people representing similar areas and thus held both small group (community advisor-specific) and larger group meetings over the course of the project. Individual groups met monthly to quarterly or less across the entire 5 years of the project and met together during annual in-person and virtual full study meetings. Representatives from all of the community advisor groups met monthly as part of the executive committee to share ideas and discuss each group’s ideas.

Community advisors’ input changed questionnaires, eligibility criteria, recruitment strategies, and other areas within the project, but these changes mostly occurred prior to study implementation, and thus any potential effect of these changes was not easily measurable. The 2 concerns presented here had measurable outcomes collected prior to and after the community advisor-guided changes to the study protocol. While community advisor engagement has a high level of face validity and in some circumstances is critical, such as interacting with specific populations within a research project, hard evidence of community advisor recommendations being associated with changes in participant research actions has been minimal. Usually, improvements in the research protocol that occur prior to any pilot or full protocol activities are impossible to quantify. Thus, the more inclusive researchers are working with community advisors from the beginning of a research pathway, the harder it is to demonstrate clear impact.

Concannon et al [14] called specifically for “evaluative research” on community advisor impact on research processes. His review did not include any quantitative outcomes from community advisor engagement. A more recent scoping review by Bird et al [21], which reviewed 1287 possible studies and reported on 14, found that all of the evaluative activities were qualitative in nature. While providing information on the process of engagement and perceived impact from the researcher and community advisor perspectives, they do not quantify that impact. Earlier studies have demonstrated improved patient recruitment and retention in 2 pediatric studies [22,23]. A more recent meta-analysis by Crocker et al [13] found 26 studies that reported on either recruitment or retention rates related to patient and public involvement in the research project. The findings related to recruitment support the construct that community advisor involvement improves recruitment (OR 1.16, 95% confidence and prediction interval 1.01‐1.34), though the total impact is small. The Crocker analysis was inconclusive concerning improvements in participant retention, though only 5 studies were available to analyze. Forsythe et al [24] reviewed 126 articles that arose from research funded by PCORI. This review describes the perceived benefits, and notably little to no risks, of including community advisors in the research process, but it does not report on any quantitative findings across these projects related to community advisor engagement. Thus, our report adds to the existing literature by providing quantitative, before-after results of changes in a research protocol based on community advisor feedback, review, and approval of the changes.

These results are from a simple before-and-after post hoc analyses of 2 areas within 1 study. Thus, it is possible that other factors accounted for the large improvements in the areas discussed earlier. These post hoc analyses are exploratory in nature. The substantial changes observed after the community advisor-guided changes to the protocol argue against the findings being purely by chance. Furthermore, the continued improved use of the ICS with nebulized rescue therapy in intervention participants enrolled after the one-time notice and ICS dispensing in December 2018, through January 2019, argues against this one-time extra contact being the primary reason for the improvement in ICS use, as opposed to the ongoing interventional activities. Nonetheless, the quasi-experimental nature of the design does not allow causation to be assessed. If the results, such as the survey response rate, can be duplicated by others using similar techniques, this would strengthen the findings.

The PREPARE study engaged a large and diverse group of community advisors throughout the study planning and conduct. Thanks to the funder’s priority of community advisor input, the costs associated with this level of effort (participant payment, meeting, and travel costs) were approved and absorbed in the overall cost of a large trial. Many research trials will not have the resources to engage community advisors to this level. The cost versus benefit of heavy community advisor engagement may not seem well balanced by all funders or all researchers. It is possible that a less intense approach to community advisor engagement could result in the same outcomes. There is no way to determine the potential impact of a smaller group of community advisors from this project. We note that our community advisor groups, particularly our patient groups and our clinical sites groups, were heavily engaged and responsive to all requests and interactions throughout the project, offering input on an ongoing basis.

### Conclusions

Multiple community advisor groups, working together to solve 2 concerns within a pragmatic clinical trial, appeared able to craft highly successful interventions that included components that may not have been suggested by the research staff alone.

## Abbreviations

ICS: inhaled corticosteroid
OR: odds ratio
PBRN: practice-based research network
PCORI: Patient-Centered Outcomes Research Institute
PREPARE: Person Empowered Asthma Relief
